# Metabolic Flexibility to Predict Lifestyle Interventions Outcomes (MEPHISTO): Protocol for Predictive Validation Study and Randomized Controlled Trial

**DOI:** 10.2196/67570

**Published:** 2025-05-08

**Authors:** Monika Ludlova, Kateřina Koudelková, Jitka Pallova, Barbora Koudelkova, Michaela Siklova, Monika Cahova, Tomas Vetrovsky, Michal Steffl, Jan Gojda

**Affiliations:** 1 Faculty of Physical Education and Sport Charles University Prague Czech Republic; 2 Clinical Research Department Kralovske Vinohrady University Hospital and Third Faculty of Medicine Charles University Prague Czech Republic; 3 Department of Pathophysiology, Centre for Research on Diabetes, Metabolism and Nutrition Third Faculty of Medicine Charles University Prague Czech Republic; 4 First Faculty of Medicine Institute of Clinical and Experimental Medicine Prague Czech Republic

**Keywords:** obesity, overweight, weight loss, physical activity, exercise, gut microbiota, metabolic flexibility, diabetes, endocrinology, validation study, randomized control trial, protocol

## Abstract

**Background:**

Weight loss is a cornerstone of obesity treatment and diabetes mellitus type 2 (T2D) prevention, but its implementation in clinical practice is limited by its perceived burden and variability in response. Personalizing interventions to increase their success rate is an unmet clinical need.

**Objective:**

Identification of predictive factors associated with successful weight loss after sequential exercise in women with obesity.

**Methods:**

The study will consist of a 2-stage analytical approach, including a predictive validation study and a 2:1 randomized cross-over controlled trial. Women aged 25-45 years with obesity (BMI>30) will be included in the study. The intervention will consist of a progressive protocol of aerobic exercise on a treadmill and a bicycle ergometer. We will measure weight loss in terms of fat mass (FM) and fat-free mass (FFM), metabolic flexibility (MetFlex) as ΔRQ (change in respiratory quotient (VCO2/VO2) between basal and insulin-stimulated state during glucose clamp), insulin sensitivity, glucose tolerance, hemoglobin A_1c_, microbiome composition, and metabolomic signatures.

**Results:**

Recruitment for the trial began in January 2024. A total of 12 participants were enrolled and randomized. Among them 6 participants have completed the first phase of the A-arm and 6 participants have completed the control period of the B-arm and their intervention is ongoing. Recruitment is ongoing. We expect the preliminary data from this study to be completed in 2026.

**Conclusions:**

This intervention will investigate whether whole body and gut MetFlex can be further explored and used as ex ante predictors of successful weight loss following exercise intervention, providing proof of concept and paving the way for personalized lifestyle interventions.

**Trial Registration:**

ClinicalTrials.gov NCT06329349; https://clinicaltrials.gov/study/NCT06329349

**International Registered Report Identifier (IRRID):**

DERR1-10.2196/67570

## Introduction

Obesity has reached pandemic proportions and is a major risk factor for type 2 diabetes (T2D), cardiovascular disease, and other metabolic disorders. Prevention progression to T2D in individuals with obesity through effective lifestyle interventions is a public health priority Substantial and sustained weight loss achieved through lifestyle modifications is a key strategy to reduce the risk of T2D and improve metabolic health [1,2]. The most effective lifestyle interventions aim to achieve a negative net energy balance through two key principles: increasing physical activity and reducing caloric intake. The health benefits associated with regular physical activity are closely linked to the systemic adaptive responses elicited by each training session [3]. Similarly, the beneficial effects of weight loss induced by hypocaloric diets depend on complex adipose tissue remodeling [4]. However, detailed analysis of clinical trial data reveals substantial interindividual variability in responses to these interventions, leading to differences in their effectiveness and health benefits [5,6].

This highlights the need for more personalized lifestyle interventions tailored to individual phenotypes. Recent studies have emphasized phenotype-based interventions as a promising strategy [7,8], particularly focusing on behavioral phenotypes and maladaptive eating patterns in patients with obesity. However, the physiological determinants that may influence weight loss success remain underexplored.

Among the influential determinants that affect the effectiveness and health outcomes of an intervention is an individual’s level of metabolic flexibility (MetFlex). MetFlex refers to the body's ability to adapt in response to changes in metabolic demands and nutrient availability [9]. Impaired MetFlex is a hallmark of obesity and T2D but can be improved by lifestyle interventions such as exercise training or caloric restriction [9-11], similar to improvements in insulin sensitivity. Despite this, MetFlex has not been systematically investigated as a potential mechanism underlying successful weight loss. Impaired MetFlex, that is the limited ability of cells or tissues to cope with excess or deficiency of energy substrates, leads to impaired mitochondrial function and excessive lipid accumulation in ectopic tissues, resulting in metabolic disorders such as T2D or metabolic syndrome.

Another potential predictor of an individual’s ability to respond to lifestyle interventions is the gut microbiota (gut microbiome and metabolome [MIME]). It has repeatedly been shown to be one of the most important sources of interindividual variability in the development of obesity [12] and also responsiveness to weight loss interventions [13]. The microbiome not only influences host physiology directly, for example through contact with immune cells, but also through the large number of metabolites produced, that is, the microbiota-derived metabolome [14]. The composition of the gut microbiota and the microbiota-derived metabolome is largely determined by the host’s diet, which is the main source of nutrients and energy for the microbiota [15]. It is therefore striking that published studies to date have yielded rather inconsistent results regarding dietary interventions to alter gut microbiota composition [16]. This discrepancy can be explained by the large variability of individual microbiomes at the start of the intervention, and also by the fact that the baseline MIME signature is a significant determinant of weight loss success.

Here we propose a comprehensive project MEPHISTO (MEtabolic flexibility to Predict Health lIfeSTyle intervention Outcomes) to investigate whether whole-body and gut MetFlex can be further explored and used as ex ante predictors of successful weight loss following exercise intervention. By identifying key physiological and microbial signatures, this research aims to provide a basis for personalized obesity treatment strategies, ultimately improving diabetes prevention efforts.

## Methods

### Design

A randomized, cross-over, controlled clinical trial will be conducted at Charles University in Prague, Czech Republic. The study will consist of a two-stage analytical approach that will be used to obtain specific results:

1. The first stage is a predictive validation study where we will compare changes in fat mass (FM)/ fat-free mass (FFM) weight loss (after an intervention - predictor variable) with baseline characteristics (independent variable) in the whole sample (n=40). The expected results are the identification of baseline characteristics associated with total and FM/FFM weight loss after the intervention.

2. The second phase is a 2:1 randomized cross-over control trial where we will investigate the effects of the progressive aerobic exercise intervention protocol on MetFlex, metabolic adaptation, and insulin sensitivity. As an exploratory outcome, the fecal metabolomic response will be analyzed.

### Participants

Women aged 25-45 years with obesity (BMI>30) are included in the study. Only female participants were selected to reduce heterogeneity, as MetFlex and energy metabolism are sex-dependent and to improve statistical power in a small sample. This selection aligns with previous studies at our research center and will allow for future data pooling. Practical considerations, including shared facilities and standardized protocols, also supported this decision.

### Inclusion and Exclusion Criteria

The inclusion and exclusion criteria are mentioned in Textbox 1.

**Inclusion and exclusion criteria.**

**Inclusion criteria**

Participants who meet the following criteria will be eligible for the study:

• BMI>30

• Age 25-45 years

**Exclusion criteria**

• Active cancer

• Diabetes (medical history, fasting glycemia >7.6, and glycemia of >11.1 on the 2-hour oral glucose tolerance test)

• Uncontrolled endocrine diseases

• Corticosteroid therapy

• Immune-suppressive therapy

• Pregnancy

• Breastfeeding

### Recruitment Process

Potential volunteers will be recruited from outpatients and previous research participants at the Department of Internal Medicine, University Hospital Kralovské Vinohrady, Prague, or through social media advertising via the university and hospital platforms. Our research participant network is only used to advertise studies, and individuals who have previously participated in interventional trials will not be included. In addition, patients from the Obesitology Centre will be considered only before their enrollment in clinical interventional programs to prevent previous interventions from influencing study outcomes.

### Allocation

After screening for eligibility, a baseline clinical examination will be performed to assess the inclusion and exclusion criteria and to ensure weight stability for 12 weeks before enrollment. Participants will then be randomized by an independent investigator to either exercise intervention arm A or exercise intervention and control arm B using a computer-generated random number table.

**Figure 1:**
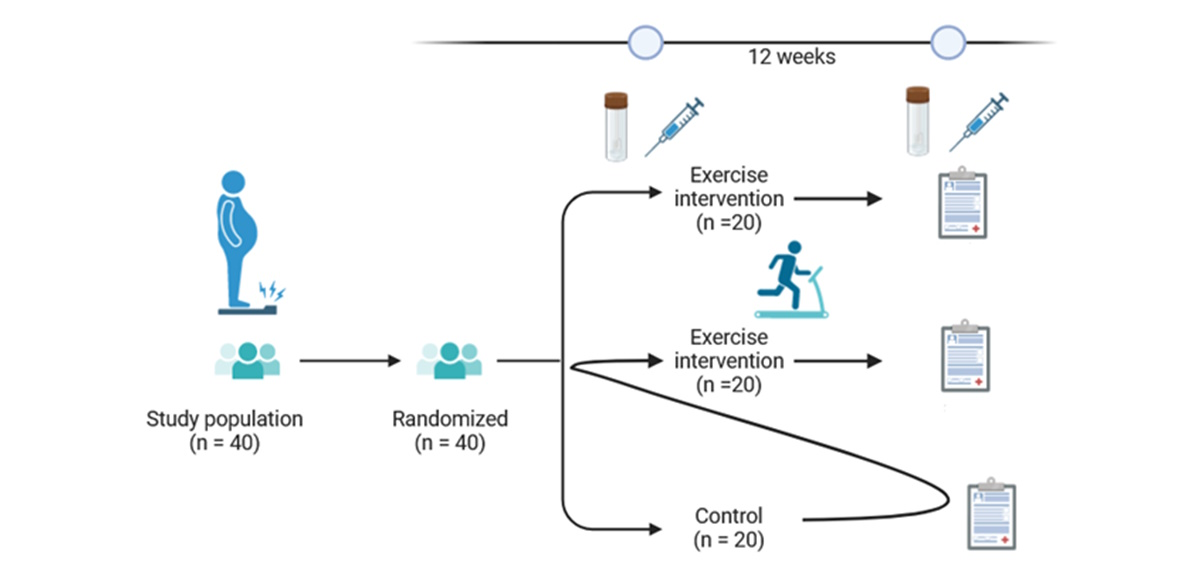
Enrollment, intervention, and evaluation project schedule.

After the baseline visit 1 examination, the A-arm will undergo 12 weeks of a progressive aerobic exercise intervention protocol, after which the clinical examination will be repeated. Participants in the B-arm will be assessed after 12 weeks of no intervention and then crossed over to the exercise intervention, after which the final clinical assessment will be carried out (Figure 1).

### Intervention

The exercise intervention is designed according to the 2021 European Association for the Study of Obesity (EASO) guidelines to maximize the effect of reducing body weight and adipose tissue [17]. These guidelines recommend 150 to 200 minutes of at least moderate-intensity aerobic exercise (Grade A). The exercise intervention will last 12 weeks, during which the study participants will perform aerobic endurance exercise 3 times a week in groups of 6 people under supervision.

The exercise will be performed as fast walking on a treadmill. The duration and intensity of the exercise will be controlled so that the energy expenditure per unit of exercise is 150 kcal in the first week, increasing by 50 kcal each week thereafter until week 6 when it reaches 400 kcal, a level that will be maintained until the end of the intervention [18]. The walking pace will be adjusted individually, based on input of indirect calorimetry, to correspond to moderate intensity physical activity (metabolic equivalent of task 3 to 6) and to achieve the required energy expenditure in the given time. A progressive increase in energy expenditure during the intervention will be therefore ensured by an appropriate combination of increasing intensity and duration of exercise. For this reason, the duration of each session will be individual, for example, 30 minutes of walking at a speed of 5 km/hour corresponds to approximately 180 kcal for a person weighing 95 kg [19].

During the intervention, participants’ compliance will be strictly monitored: if a session is missed, it will be made up as soon as possible. If more than one session is missed (eg, due to illness), the intervention will be extended to make up for the missed sessions, but not more than 2 weeks. Only participants who complete at least 90% of the training sessions will be included in the analysis (so they can skip a maximum of 3 sessions).

### Primary Outcome Measures

Stage 1: the relationship between FM/FFM weight loss and baseline characteristics of MetFlex, calculated as the change in respiratory quotient (ΔRQ).

Stage 2: change in MetFlex, defined as the ΔRQ between basal and insulin-stimulated state during glucose clamp examination, and change in insulin sensitivity (glucose infusion rate).

### Secondary Outcome Measures

Changes in glucose tolerance (glucose level on the 2-hour oral glucose tolerance test), hemoglobin A_1c_, and insulin sensitivity (glucose infusion rate) following the intervention will be evaluated following the intervention.

### Exploratory Outcome Measures

Changes in microbiome composition and metabolomic signatures will also be assessed following the intervention.

### Data Collection

#### Anthropometry

The anthropometric measures used were weight, height, bioimpedance analysis (TANITA MC-980U Plus Multi-Frequency Segmental Body Composition Analyzer, manufacturer, state), DXA (Hologic’s Horizon DXA Platform), and plethysmography (BOD POD’s Air Displacement Plethysmography Cosmed).

#### Indirect Calorimetry

The resting gas exchange will be measured using a canopy hood ventilated system with a metabolic cart (Quark RMR, Cosmed). Standard air, gas, and flow calibrations will be performed before each measurement. Important deviations may limit the reliability of measurements, to correct for this postcalorimetric simulation with high-precision mass flow regulators (EL-Flow Prestige, Bronkhorst high-tech B.V., NL) will be done after each measurement with a defined gas mixture. VO_2_, VCO_2,_ and nitrogen loss per 24 hours will be used to calculate basal substrate utilization of carbohydrates, fat, and protein.

#### Oral Glucose Tolerance Test

A standard 75 g oral glucose tolerance test will be performed with blood sampling at 0, 30, 60, 90, and 120 minutes. The test will assess dynamic changes in substrate metabolism, including ΔRQ shifts over time (120–60–0 min) and changes in circulating free fatty acids (ΔFFA), glucose, and insulin levels in response to the glucose load.

#### Hyperinsulinemic-Euglycemic Clamp

A 2-step hyperinsulinemic-euglycemic clamp will be conducted over 200 minutes, with insulin infusion rates of 10 and 40 mIU/m², each maintained for 100 minutes. This will be followed by a 100-minute tapering period of glucose infusion after insulin discontinuation. The primary outcomes will include ΔRQ (at 300–250–200–100–0 min), free fatty acid suppression (ΔFFA), and glucose infusion rate (ΔGIR) as markers of insulin sensitivity and MetFlex.

#### Maximal Oxygen Uptake Test

A graded maximal exercise test will be performed on a bicycle ergometer (Ergoline GmbH) using a ramp protocol with an incremental workload increase of 15 W/min. Gas exchange parameters will be measured continuously via spiroergometry (Quark RMR), and electrocardiogram monitoring will be conducted throughout the test. The primary outcomes (maximal oxygen uptake [VO₂max], heart rate, and peak power output), will be used to individualize the exercise intervention protocol.

#### Diet Record

Each participant will provide a 3-day diet record before the intervention or control period, once during the period, and then after the completion of the intervention or control. Nutrixo (Arcai Health) software with validated food composition databases will be used to calculate dietary intakes.

#### Metagenomics Analysis of Gut Microbiota

The microorganism populations present in the stool samples will be assessed by shallow shotgun sequencing using NovaSeq ox NextSeq Illumina platform targeting sequencing depth of approximately 1 million reads or samples. Demultiplexing, BAM and FASTQ file generation will be performed using the Picard suite (Broad Institute). Quality-filtered metagenomes will be taxonomically profiled using MetaPhlAn3 (Metagenomic Phylogenetic Analysis) with default parameters.

#### Untargeted Analysis of Serum Metabolome

Serum metabolite spectrum will be determined using NMR-based methods. Serum samples will be analyzed on Bruker AVANCE III 600 MHz and 700 MHz spectrometers according to the standard protocols. Metabolites will be identified by comparison with spectral databases (BBIOREFCODE, Chenomx, HMDB [Human Metabolome Database]) and published assignments. Liquid chromatography–mass spectrometry analysis in serum extracts will be performed using previously optimized procedures including extraction, separation by liquid chromatography (Agilent 1200), and detection by Bruker MS spectrometer micrOTOF-Q III. Suitable conditions will allow identification per exact mass, retention time, isotopic pattern, and fragmentations. For this purpose, Bruker software and MZmine 2.17 will be used.

### Data Analysis and Statistical Considerations

To assess the effectiveness of the weight loss intervention and identify key predictors of response, such as microbiome, metabolomic profiles, and demographics, we will use generalized mixed effects models. These models account for both fixed effects (the intervention and relevant covariates) and individual variability, providing a robust analysis of the impact of the intervention. In addition, we will develop elastic net-based predictive models, complemented by machine learning algorithms, to stratify individuals into intervention groups, with the secondary aim of identifying responders. These models will be subjected to rigorous validation, including cross-validation or bootstrapping, to ensure their accuracy and generalizability. This approach aims not only to determine the efficacy of the intervention but also to improve personalized treatment by accurately classifying individuals based on their unique profiles, thereby advancing the field of personalized medicine. The analyses will be performed using IBM SPSS Statistics and R statistical packages.

### Sample Size

Sample size considerations will be based on the power to address the primary hypothesis. The main predictor variables for the sample are ΔRQ (200-0) and glucose disposal (mg/kg/min). In recent observations from our TRIEMA (Treatment of Insulin Resistance: a Personalized Approach) study (NCT03710850), glucose disposal was 6.6 (SD 2.1), while ΔRQ was 0.05 (SD 0.04). The dependent variable, the expected weight loss after the intervention, is 4 (SD 5) kg [20]. The 30-subject study will have 80% power to detect a difference in weight loss of 4 kg (which is a clinically significant 5% weight loss for a woman weighing 80 kg at a difference of ΔRQ=0.07 (calculated using the PWRSS library in the R environment), at a probability level of *α*=.05. We plan to enroll 40 women to allow for attrition to the end of the intervention without risk of losing statistical power.

### Major Confounders

It is known from the literature that people who start a structured exercise program may experience behavioral compensation in the form of increased food intake [21] and reduced habitual physical activity in everyday life [22,23]. Therefore, in our study, we will monitor participants’ caloric intake using repeated 24-hour recalls and physical activity using an accelerometer. Caloric intake and habitual physical activity will always be assessed (1) in the week before the exercise intervention, (2) in the 6th week of the intervention, that is, after reaching the target energy expenditure, and (3) in the last 12th week of the intervention. In each of these weeks, caloric intake is assessed using 24-hour menu records from 3 specific days (the day of exercise, a weekday without exercise, and the weekend), using an application that works with validated food composition tables [24]. The caloric intake for each of the 3 weeks will be calculated from these 3 days.

Physical activity will be assessed in each of the specified weeks using the Axivity AX3 accelerometer. The accelerometer will be attached to the participant’s wrist and worn within 24 hours for 7 full days. The data from the accelerometer will be processed using the GGIR library in R and the volume (average acceleration) and intensity (intensity gradient) of physical activity [25] will be calculated for each of the 3 weeks.

Compensation will be calculated as the difference between caloric intake or physical activity during the intervention (average of weeks 6 and 12) and before the intervention.

### Feasibility Consideration

The team has extensive experience in conducting clinical trials and all methods are routinely available in the team members’ facilities. Therefore, we do not anticipate any feasibility issues. We anticipate difficulties in recruitment and enrollment due to the challenging protocol and strict inclusion and exclusion criteria and anticipate a buffer time in the timetable for up to double the recruitment of participants. In case of an emergency situation that would make it impossible to carry out the clinical trial (such as the experience during the COVID-19 pandemic), we plan to work within work package 2 with databases and biobank material from previous studies that would allow the project outputs.

### Ethical Considerations

The institutional review board of the University Hospital (EK-VP/28/1/2022) and the Faculty of Physical Education and Sport of Charles University (218/2023) reviewed the protocol before recruitment, and all participants will sign an informed consent form before enrollment. The study will be conducted in accordance with the Declaration of Helsinki and GCP.

The study will be conducted in accordance with the Declaration of Helsinki and Good Clinical Practice (GCP). The protocol was reviewed by the institutional ethics committee of University Hospital Kralovske Vinohrady and the Faculty of Physical Education and Sport Charles University.

## Results

Recruitment for the trial began in January 2024. A total of 12 participants were enrolled and randomized. Among them, 6 participants have completed the first phase of the A-arm and 6 participants have completed the control period of the B-arm and their intervention is ongoing. Recruitment is ongoing. We expect the preliminary data from this study to be completed in 2026. This clinical trial protocol was developed based on the SPIRIT (Standard Protocol Items: Recommendations for Interventional Trials) 2013 Statement [26] (Multimedia Appendix 1).

## Discussion

This randomized trial will assess the strength of predictive factors associated with successful weight loss after an exercise intervention in women with obesity. By focusing on both whole-body and gut MetFlex, the study aims to elucidate key mechanisms that contribute to individual variability in weight loss outcomes. The findings have the potential to lead to the development of more personalized and effective weight loss treatments, filling a critical gap in current obesity and diabetes management.

Several key decisions were made in the design of this clinical trial to overcome limitations in existing literature and reduce potential bias. One such decision is the use of a cross-over trial design, which enhances the robustness of the study by allowing participants to serve as their own controls. This approach helps reduce interindividual variability and provides stronger evidence of the causal relationship between the interventions and the observed outcomes.

Lifestyle interventions, particularly those combining dietary and exercise components, are as essential for managing and potentially reversing diabetes progression [27,28]. Substantial evidence supports the role of sustained weight loss in improving insulin sensitivity, glycemic control, and other metabolic outcomes. However, the heterogeneity in response to such interventions highlights a critical need for personalization [29,30]. Individuals vary widely in their ability to lose weight and maintain that loss, which is often attributed to differences in MetFlex, the gut microbiome, and behavioral adaptations [31]. An additional strength of this study is the comprehensive monitoring of participants’ dietary intake and physical activity throughout the intervention. By accounting for compensatory behaviors, such as changes in caloric intake or reduced physical activity outside of exercise sessions, we aim to provide a clearer understanding of how these factors contribute to weight loss variability.

However, some limitations need to be considered. The strict eligibility criteria may make recruitment challenging, potentially limiting generalizability to the broader population of women with obesity. Furthermore, while compensatory behaviors are monitored, other factors such as psychological and social influences could still impact outcomes.

In conclusion, this trial will provide important insights into the role of MetFlex and the gut microbiome in predicting successful weight loss. These findings could lead to more personalized interventions in clinical practice, ultimately improving adherence and outcomes in obesity and diabetes management.

## Appendix

Multimedia Appendix 1 SPIRIT checklist.

## Abbreviations

EASO: European Association for the Study of Obesity
FFM: fat-free mass
FM: fat mass
HMDB: Human Metabolome Database
MEPHISTO: Metabolic flexibility to Predict Health Lifestyle Intervention Outcomes
MetaPhlAn3: Metagenomic Phylogenetic Analysis
MetFlex: metabolic flexibility
MIME: gut microbiome and metabolome
SPIRIT: Standard Protocol Items: Recommendations for Interventional Trials
T2D: type 2 diabetes
VO₂max: maximal oxygen uptake
ΔFFA: changes in circulating free fatty acids
ΔGIR: change in glucose infusion rate
ΔRQ: change in respiratory quotient

## Data Availability

The datasets generated or analyzed during this study are available from the corresponding author on reasonable request.
