# Supplementary material for: Metabolic Flexibility to Predict Lifestyle Interventions Outcomes (MEPHISTO): Protocol for Predictive Validation Study and Randomized Controlled Trial
Source: JMIR Res Protoc. 2025 May 8;14:e67570. doi: 10.2196/67570 (PMC12099273; doi:10.2196/67570)
Supplement: Multimedia Appendix 1 [file resprot_v14i1e67570_app1.pdf]

SPIRIT 2013 Checklist – Appendix 1: MEPHISTO Protocol

This document contains the completed SPIRIT 2013 checklist for the study protocol titled 'Metabolic Flexibility to Predict Lifestyle Interventions Outcomes (MEPHISTO)'. The checklist ensures that all essential elements of a clinical trial protocol are addressed.

Title (1)

Description: Descriptive title identifying the study design, population, interventions, and, if applicable, trial acronym

Addressed in Protocol: Metabolic Flexibility to Predict Lifestyle Interventions Outcomes (MEPHISTO): Protocol for Predictive Validation Study and Randomized Controlled Trial

Trial registration (2a)

Description: Trial identifier and registry name

Addressed in Protocol: Registered: NCT06329349

Trial registration (2b)

Description: All items from the WHO Trial Registration Data Set

Addressed in Protocol: Included in ClinicalTrials.gov entry

Protocol version (3)

Description: Date and version identifier

Addressed in Protocol: Version 1.0, April 2025

Funding (4)

Description: Sources and types of support

Addressed in Protocol: Funded by the Ministry of Health of the Czech Republic (NU23-01-00509) and Charles University (grant 74524)

Roles and responsibilities (5a-d)

Description: Names, affiliations, roles; sponsor info; funders' role; coordinating structure

Addressed in Protocol: Authors and contributors listed with affiliations and roles in protocol. Sponsors have no role in design, analysis, or publication decisions.

Background and rationale (6a)

Description: Justification for the trial and relevant studies

Addressed in Protocol: Detailed in Introduction, citing current gaps in personalized interventions based on MetFlex and microbiome.

Choice of comparators (6b)

Description: Explanation for control/comparator

Addressed in Protocol: Control is a non-intervention period in the crossover design.

Objectives (7)

Description: Specific objectives or hypotheses

Addressed in Protocol: To identify predictors of weight loss success, and evaluate MetFlex as a key mechanism.

Trial design (8)

Description: Type of trial, allocation ratio, framework

Addressed in Protocol: Two-stage design: predictive validation and 2:1 randomized crossover controlled trial.

Study setting (9)

Description: Location(s) and settings

Addressed in Protocol: University Hospital Kralovské Vinohrady and Charles University, Prague, Czech Republic.

Eligibility criteria (10)

Description: Inclusion and exclusion criteria

Addressed in Protocol: Women aged 25–45, BMI >30; specific exclusions listed (e.g., diabetes, pregnancy).

Interventions (11a-d)

Description: Details to allow replication, adherence strategies, modifications, co-interventions

Addressed in Protocol: 12-week supervised aerobic protocol with progressive energy expenditure; compliance tracked, adherence measures described.

Outcomes (12)

Description: Primary, secondary, exploratory outcomes

Addressed in Protocol: ΔRQ, insulin sensitivity, microbiome and metabolomics changes.

Participant timeline (13)

Description: Schedule of enrolment, intervention, assessment

Addressed in Protocol: Outlined in Figure 1 of protocol.

Sample size (14)

Description: Estimates and assumptions

Addressed in Protocol: Sample size of 40 based on ΔRQ, insulin sensitivity, weight loss; powered for 30 completers.

Recruitment (15)

Description: Strategies for enrolment

Addressed in Protocol: Via clinic network, prior research participants, and university/hospital social media outreach.

Sequence generation (16a)

Description: Method of randomization

Addressed in Protocol: Computer-generated random sequence by independent investigator.

Allocation concealment (16b)

Description: Method for concealment

Addressed in Protocol: Independent investigator assigns participants using sealed envelopes.

Implementation (16c)

Description: Roles in allocation

Addressed in Protocol: Allocation by staff uninvolved in outcome assessments.

Blinding (17a,b)

Description: Who is blinded and unblinding procedures

Addressed in Protocol: Outcome assessors blinded; no blinding of participants due to nature of intervention.

Data collection methods (18a,b)

Description: Plans for data collection, retention

Addressed in Protocol: Standardized instruments and protocols; 3-day diet records, accelerometers for activity, validated measures used.

Data management (19)

Description: Entry, coding, storage

Addressed in Protocol: Data stored securely, double-entry, monitored for range and completeness.

Statistical methods (20a-c)

Description: Analyses, handling missing data

Addressed in Protocol: Mixed models, machine learning, imputation methods; described in detail.

Data monitoring (21a,b)

Description: DMC composition, interim analyses

Addressed in Protocol: No DMC due to pilot scale; no interim analyses planned.

Harms (22)

Description: Plans for AE reporting

Addressed in Protocol: All adverse events monitored and reported to IRB.

Auditing (23)

Description: Trial audits

Addressed in Protocol: Internal audits by principal investigator and sponsor monitors.

Ethics approval (24)

Description: Plans for IRB approval

Addressed in Protocol: Approved by ethics boards of University Hospital KV and Charles University.

Protocol amendments (25)

Description: Communication plans

Addressed in Protocol: Amendments communicated to ethics committees and updated on ClinicalTrials.gov.

Consent (26a,b)

Description: Informed consent procedures

Addressed in Protocol: Written consent obtained by trained staff; no biological samples stored for future use.

Confidentiality (27)

Description: Data protection measures

Addressed in Protocol: Personal data anonymized, access restricted.

Declaration of interests (28)

Description: Investigators' COIs

Addressed in Protocol: No competing interests declared.

Access to data (29)

Description: Who has access

Addressed in Protocol: Investigators have access; no restrictive agreements.

Ancillary care (30)

Description: Post-trial care

Addressed in Protocol: No ancillary care provided; participants informed of health findings.

Dissemination (31a-c)

Description: Plans to share results

Addressed in Protocol: Results to be shared via publications, conferences, and ClinicalTrials.gov.

Informed consent materials (32)

Description: Model forms

Addressed in Protocol: Provided to ethics committee; available upon request.

Biological specimens (33)

Description: Plans for biobanking

Addressed in Protocol: Not applicable; specimens not stored for future use.
